# Complexes of Ghrelin GHS-R1a, GHS-R1b, and Dopamine D_1_ Receptors Localized in the Ventral Tegmental Area as Main Mediators of the Dopaminergic Effects of Ghrelin

**DOI:** 10.1523/JNEUROSCI.1151-21.2021

**Published:** 2022-02-09

**Authors:** Gemma Navarro, William Rea, César Quiroz, Estefanía Moreno, Devan Gomez, Cody J. Wenthur, Vicent Casadó, Lorenzo Leggio, Matthew C. Hearing, Sergi Ferré

**Affiliations:** ^1^Department of Biochemistry and Molecular Biology, Faculty of Pharmacy and Food Sciences, University of Barcelona, Barcelona, 08028, Spain; ^2^Integrative Neurobiology Section, Molecular Targets and Medications Discovery Branch, National Institute on Drug Abuse, Intramural Research Program, National Institutes of Health, Baltimore, Maryland 21224; ^3^Department of Biochemistry and Molecular Biomedicine and Institute of Biomedicine, Faculty of Biology, University of Barcelona, 08028 Barcelona, Spain; ^4^Department of Biomedical Sciences, Marquette University, Milwaukee, Wisconsin 53233; ^5^School of Pharmacy, University of Wisconsin, Madison, Wisconsin 53705; ^6^Clinical Psychoneuroendocrinology and Neuropsychopharmacology Section, Translational Addiction Medicine Branch, National Institute on Drug Abuse Intramural Research Program, and National Institute on Alcohol Abuse and Alcoholism Division of Intramural Clinical and Biological Research, National Institutes of Health, Baltimore, Maryland 21224

**Keywords:** dopamine, dopaminergic neurons, ghrelin, microdialysis, receptor heteromers, ventral tegmental area

## Abstract

Ghrelin receptor, also known as growth hormone secretagogue receptor (GHS-R1a), is coexpressed with its truncated isoform GHS-R1b, which does not bind ghrelin or signal, but oligomerizes with GHS-R1a, exerting a complex modulatory role that depends on its relative expression. D_1_ dopamine receptor (D1R) and D5R constitute the two D_1_-like receptor subtypes. Previous studies showed that GHS-R1b also facilitates oligomerization of GHS-R1a with D1R, conferring GHS-R1a distinctive pharmacological properties. Those include a switch in the preferred coupling of GHS-R1a from Gq to Gs and the ability of D1R/D5R agonists and antagonists to counteract GHS-R1a signaling. Activation of ghrelin receptors localized in the ventral tegmental area (VTA) seems to play a significant role in the contribution of ghrelin to motivated behavior. In view of the evidence indicating that dopaminergic cells of the VTA express ghrelin receptors and D5R, but not D1R, we investigated the possible existence of functional GHS-R1a:GHS-R1b:D5R oligomeric complexes in the VTA. GHS-R1a:GHS-R1b:D5R oligomers were first demonstrated in mammalian transfected cells, and their pharmacological properties were found to be different from those of GHS-R1a:GHS-R1b:D1R oligomers, including weak Gs coupling and the ability of D1R/D5R antagonists, but not agonists, to counteract the effects of ghrelin. However, analyzing the effect of ghrelin in the rodent VTA on MAPK activation with *ex vivo* experiments, on somatodendritic dopamine release with *in vivo* microdialysis and on the activation of dopaminergic cells with patch-clamp electrophysiology, provided evidence for a predominant role of GHS-R1a:GHS-R1b:D1R oligomers in the rodent VTA as main mediators of the dopaminergic effects of ghrelin.

**SIGNIFICANCE STATEMENT** The activation of ghrelin receptors localized in the ventral tegmental area (VTA) plays a significant role in the contribution of ghrelin to motivated behavior. We present evidence that indicates these receptors form part of oligomeric complexes that include the functional ghrelin receptor GHS-R1a, its truncated nonsignaling isoform GHS-R1b, and the dopamine D_1_ receptor (D1R). The binding of ghrelin to these complexes promotes activation of the dopaminergic neurons of the VTA by activation of adenylyl cyclase–protein kinase A signaling, which can be counteracted by both GHS-R1a and D1R antagonists. Our study provides evidence for a predominant role of GHS-R1a:GHS-R1b:D1R oligomers in rodent VTA as main mediators of the dopaminergic effects of ghrelin.

## Introduction

The orexigenic hormone ghrelin acts as an internal signal for animals to engage in food-directed behaviors (Silver and Balsam, 2010; Mason et al., 2014; Al Massadi et al., 2019). It is mainly produced by the stomach oxyntic cells during anticipated mealtimes, and its blood levels decrease after meals (Silver and Balsam, 2010). The hypothalamic arcuate nucleus is a major site of expression for ghrelin receptors in the brain, specifically in the cells that synthesize the neuropeptide agouti-related peptide. Ghrelin reaches the arcuate nucleus by its proximity to the median eminence, where the blood–brain barrier is more permissive and allows the passage of peptides (for review, see Ferré, 2017). Agouti-related peptide neurons project to several hypothalamic nuclei, as well as to the ventral tegmental area (VTA), and their activation promotes feeding (Aponte et al., 2011; Krashes et al., 2011; Betley et al., 2013). Ghrelin receptors are also expressed in other brain regions, including the hippocampus, substantia nigra, and VTA (Zigman et al., 2006). Several studies suggest that the activation of the VTA dopaminergic system plays a significant role in the motivational function of ghrelin (Abizaid et al., 2006; Jerlhag et al., 2007; Skibicka et al., 2011; Al Massadi et al., 2019). The mechanisms proposed to be involved in such activation include direct peripheral transit of ghrelin to the VTA or indirect activation through circuit-level effects of ghrelin from either peripheral or central origin (see Discussion).

The ghrelin receptor is a G-protein-coupled receptor (GPCR) also known as growth hormone secretagogue receptor (GHSR), or GHS-R1a. Cells expressing GHS-R1a also express GHS-R1b, a truncated isoform lacking transmembrane domains 6 and 7. Although ghrelin is the canonical ligand for GHS-R1a, ghrelin does not bind and does not signal through GHS-R1b. However, GHS-R1b oligomerizes with GHS-R1a, exerting an expression level-dependent modulatory role (Navarro et al., 2016). Low relative GHS-R1b expression facilitates GHS-R1a function by promoting its trafficking to the plasma membrane, while a high relative GHS-R1b expression inhibits GHS-R1a function by exerting a negative allosteric effect on GHS-R1a signaling (Mary et al., 2013; Navarro et al., 2016).

Another functional role of GHS-R1b is facilitating the oligomerization of GHS-R1a with other GPCRs, specifically with the dopamine D_1_ receptor (D1R), which was shown both in mammalian transfected cells and in striatal cells in culture (Navarro et al., 2016). Among the pharmacological properties of the GHS-R1a:GHS-R1b:D1R oligomer, ghrelin promoted adenylyl cyclase (AC) activation, indicating a switch in the usually preferred Gq coupling of GHS-R1a to the preferred Gs coupling of D1R (Navarro et al., 2016). Other studies in mammalian transfected cells and hippocampal neurons have also provided evidence for the ability of GHS-R1a to heteromerize with D1R without the involvement of GHS-R1b, in which case ghrelin did not produce AC activation, while D1R could signal by coupling to Gq (Jiang et al., 2006; Kern et al., 2015; Casanovas et al., 2021). Immunohistochemical experiments in transgenic mice that express GFP in cells also expressing GHS-R1a indicated a prominent colocalization of GHS-R1a and D1R in the hippocampus, cortex, and midbrain, in the substantia nigra and VTA (Jiang et al., 2006; Kern et al., 2015). Therefore, we investigated whether GHS-R1a:D1R complexes localized in the VTA constitute a significant population of GHS-R1a receptors mediating the dopaminergic effects of ghrelin.

Since some studies indicate that D5R is the predominant D_1_-like receptor localized in mesencephalic dopaminergic cells (Ciliax et al., 2000; Khan et al., 2000), we aimed first at analyzing the possible existence of oligomers of GHS-R1a, GHS-R1b, and D5R in mammalian transfected cells and comparing their pharmacological properties to those of GHS-R1a:GHS-R1b:D1R oligomers. We then evaluated the possible existence of these oligomeric complexes in the rodent VTA as well as their role in the dopaminergic effects of ghrelin. Using different *in vitro* and *in vivo* approaches, we obtained evidence for a predominant role of GHS-R1a:GHS-R1b:D1R oligomers, rather than R1a:GHS-R1b:D5R oligomers, in rodent VTA as the main mediators of the dopaminergic effects of ghrelin.

## Materials and Methods

### Vectors, fusion proteins, cell culture, and transfection.

Human cDNAs for GHS-R1a, GHSR1b, and D5R, cloned into pcDNA3.1, were amplified without their stop codons using sense and antisense primers harboring EcoRI and KpnI sites to clone D5R in the pRLuc-N1 vector (pRLuc-N1; PerkinElmer Life Sciences) and GHS-R1a or GHS-R1b to the pEYFP-N1 vector (enhanced yellow variant of GFP; Clontech) or EcoRI and KpnI sites to clone GHS-R1a in a GFP^2^-containing vector (p-GFP^2^; Packard BioScience). Amplified fragments were subcloned to be in frame with restriction sites of pRLuc-N1, pEYFP-N1, or p-GFP^2^ vectors to provide plasmids that express proteins fused to RLuc, yellow fluorescent protein (YFP), or GFP^2^ on the C-terminal end (D5R-RLuc, GHS-R1a-YFP, GHS-R1b-YFP, and GHS-R1a-GFP^2^). HEK293T cells were maintained at 37°C in an atmosphere of 5% CO_2_. They were grown in DMEM (Thermo Fisher Scientific) supplemented with 2 mm l-glutamine, 100 µg/ml sodium pyruvate, 100 U/ml penicillin/streptomycin, minimum Eagle's medium nonessential amino acid solution (1:100), and 5% (v/v) heat-inactivated FBS (all supplements were from Thermo Fisher Scientific). HEK293T cells were transiently transfected with the corresponding protein cDNA by the polyethylenimine (PEI) (Sigma-Aldrich) method. Cells were incubated for 4 h with the corresponding cDNA together with PEI (5.47 mm in nitrogen residues) and 150 mm NaCl in a serum-starved medium. After 4 h, the medium was changed to a fresh complete culture medium. Forty-eight hours after transfection, cells were washed twice in quick succession in HBSS with 10 mm glucose, detached, and resuspended in the same buffer, and used for bioluminescence resonance energy transfer (BRET), sequential resonance energy transfer (SRET), and cAMP accumulation or ERK1/2 phosphorylation experiments.

### Resonance energy transfer-based assays.

For BRET experiments, HEK293T cells were transiently cotransfected with a constant cDNA encoding for receptor-RLuc and with increasing amounts of cDNA corresponding to receptor-YFP (BRET^1^) or receptor-GFP^2^ (BRET^2^). To control the cell number, the sample protein concentration was determined using a Bradford assay kit (BIO-RAD) using bovine serum albumin dilutions as standards. To quantify fluorescence proteins, cells (20 µg protein) were distributed in 96-well microplates (black plates with a transparent bottom) and the fluorescence was read in a Fluostar Optima fluorimeter (BMG Labtech) equipped with a high-energy xenon flash lamp using a 10 nm bandwidth excitation filter at 410 nm for receptor-GFP^2^ reading or 485 nm for receptor-YFP reading. Receptor fluorescence expression was determined as fluorescence of the sample minus the fluorescence of cells expressing receptor-Rluc alone. For BRET measurements, the equivalent of 20 µg of cell suspension was distributed in 96-well white microplates with white bottoms (Corning 3600, Corning) and 5 μm coelenterazine H (for the YFP acceptor) or DeepBlueC (for the GFP2 acceptor; Thermo Fisher Scientific) were added. Using DeepBlueC or coelenterazine H as substrates results in respective 410- and 485 nm emissions from RLuc, which allows the respective selective energy transfer to GFP^2^ and YFP (Carriba et al., 2008). One minute after adding coelenterazine H or immediately after addition of DeepBlueC, BRET was determined using a Mithras LB 940 reader (Berthold Technologies), which allows the integration of the signals detected in the short-wavelength filter at 485 nm and the long-wavelength filter at 530 nm when YFP is the acceptor or the short-wavelength filter at 410 nm and the long-wavelength filter at 510 nm when GFP^2^ is the acceptor. To quantify receptor-RLuc expression, luminescence readings were performed after 10 min of adding 5 μm coelenterazine H regardless of the acceptor used. Net BRET is defined as [(long wavelength emission)/(short wavelength emission)] – Cf, where Cf corresponds to [(long wavelength emission)/(short wavelength emission)] for the RLuc construct expressed alone in the same experiment. For SRET experiments (Carriba et al., 2008), HEK293T cells were transiently cotransfected with constant amounts of cDNAs encoding both the receptor fused to RLuc or GFP^2^ and with increasingly amounts of cDNA corresponding to the receptor fused to YFP. Using aliquots of transfected cells (20 µg of protein), the following different determinations were performed in parallel: quantification of protein-YFP expression; and quantification of protein-RLuc expression as described above. For SRET, cells were distributed in 96-well microplates, and 5 μm DeepBlueC was added. The SRET signal was collected using a Mithras LB 940 reader with detection filters for short wavelength (410 nm) and long wavelength (530 nm). By analogy with BRET, net SRET is defined as [(long wavelength emission)/(short wavelength emission)] – Cf, where Cf corresponds to long wavelength emission/short wavelength emission for cells expressing protein-RLuc and protein-GFP^2^. Linear unmixing was done for SRET quantification, taking into account the spectral signature to separate the two fluorescence emission spectra (Zimmermann et al., 2002). BRET and SRET are expressed as milliBRET units (mBU) and milliSRET units (mSU); net BRET and net SRET X 1000).

### cAMP accumulation assay.

Homogeneous time-resolved fluorescence energy transfer assays were performed using the Lance Ultra cAMP kit (PerkinElmer Life Sciences). The optimal cell density was first established for an appropriate fluorescent signal by measuring the time-resolved fluorescence resonance energy transfer (FRET) signal as a function of forskolin concentration using different cell densities. Forskolin dose–response curves were related to the cAMP standard curve to establish which cell density provides a response that covers most of the dynamic range of the cAMP standard curve. HEK293T cells growing in medium containing 50 μm zardaverine, a phosphodiesterase inhibitor, were incubated for 15 min before either ghrelin or vehicle was added. Fluorescence at 665 nm was analyzed on a PHERAstar Flagship microplate reader equipped with a homogeneous time-resolved fluorescence energy transfer optical module (BMG Labtech).

### Determination of phosphorylated ERK1/2 in HEK293T cells or in VTA slices.

Transfected HEK293T cells were cultured in serum-free medium for 16 h before the addition of any ligand. Cells or rat VTA slices were treated or not with the indicated ligands for the indicated time (see corresponding figures) and were lysed by the addition of 300 μl of ice-cold lysis buffer (50 mm Tris-HCl, pH 7.4, 50 mm NaF, 150 mm NaCl, 45 mm β-glycerophosphate, 1% Triton X-100, 20 μm phenyl-arsine oxide, 0.4 mm NaVO_4_, and a protease inhibitor mixture). Cellular debris was removed by centrifugation at 13,000 × *g* for 5 min at 4°C, and the protein was quantified by the bicinchoninic acid method using BSA dilutions as the standard. Phosphorylated proteins were then determined by Western blot, using a mouse anti-phospho-ERK1/2 antibody (1:2500; Sigma-Aldrich) and rabbit anti-total-ERK1/2 antibody (1:40,000; Sigma-Aldrich) to quantify phospho-ERK1/2. Bands were visualized by the addition of a mixture of IRDye 800 (anti-mouse) antibody (1:10,000; Sigma-Aldrich) and IRDye 680 (anti-rabbit) antibody (1:10,000; Sigma-Aldrich) and scanned by an Odyssey infrared scanner (LI-COR). Band densities were quantified using the scanner software exported to Excel (Microsoft). The level of phosphorylated proteins was normalized for differences in loading using the total (phosphorylated plus nonphosphorylated) protein band intensities.

### Animals.

All animals used in the study were maintained in accordance with the National Institutes of Health *Guide for the Care and Use of Laboratory Animals*, and the animal research conducted to perform this study was reviewed and approved by the National Institute on Drug Abuse (NIDA) Intramural Research Program (IRP) Animal Care and Use Committee (protocol no. 18-MTMD-13), the Catalan Ethical Committee for Animal Use (protocol CEAA/DMAH no. 4049 and no. 5664) and the Institutional Animal Care and Use Committee at Marquette University (protocols AR-290 and AR-291). Male Sprague Dawley rats (from the in-house colony of the Faculty of Biology, University of Barcelona or Charles River Laboratories) were used to obtain VTA slices and for microdialysis experiments. Male GHS-R CRISPR/Cas9 knock-out (KO) Wistar rats and wild-type (WT) littermates (Zallar et al., 2019; in-house colony at NIDA IRP) were also used in microdialysis experiments. Pitx3-eGFP(+) mice (Zhao et al., 2004), provided by Kevin Wickman (University of Minnesota, Minneapolis, MN) with permission from Meng Li (Cardiff University, Cardiff, UK), and C57BL6/J mice from the in-house colony at Marquette University (Milwaukee, WI) were used for electrophysiology experiments.

### Rat VTA slice preparation.

Male Sprague Dawley rats (2 months old) were killed by decapitation under 4% isoflurane anesthesia, and brains were rapidly removed, placed in ice-cold oxygenated (O_2_/CO_2_, 95%/5%) Krebs–HCO_3_^–^ buffer (in mm: 124 NaCl, 4 KCl, 1.25 KH_2_PO_4_, 1.5 MgCl_2_, 1.5 CaCl_2_, 10 glucose, and 26 NaHCO_3_, pH 7.4), and sliced at 4°C using a brain matrix (Zivic Instruments). VTA slices (500 µm thick) were dissected at 4°C in Krebs–HCO_3_^–^ buffer; each slice was transferred into a 12-well plate with Corning Netwell inserts containing 2 ml of ice-cold Krebs–HCO_3_^–^ buffer. The temperature was raised to 23°C, and, after 30 min, the medium was replaced by 2 ml of fresh buffer (23°C). Slices were incubated under constant oxygenation (O_2_/CO_2_, 95%/5%) at 30°C for 4 h in an Eppendorf Thermomixer (5′), and the medium was replaced by fresh buffer and incubated for 30 min before the addition of any ligand. After incubation, the solution was discarded, and slices were frozen on dry ice and stored at 80°C until ERK1/2 phosphorylation was determined.

### *In vivo* microdialysis.

Male Sprague Dawley rats, GHS-R CRISPR/Cas9 KO Wistar rats, or their WT littermates (3 months old) were used. Experiments were performed during the light cycle. Rats were deeply anesthetized with 3 ml/kg Equithesin (4.44 g of chloral hydrate, 0.972 g of Na pentobarbital, 2.124 g of MgSO_4_, 44.4 ml of propylene glycol, 12 ml of ethanol, and distilled H_2_O up to 100 ml of the final solution; NIDA Pharmacy) and implanted unilaterally in the VTA (coordinates in millimeters from bregma with a 10° angle in the coronal plane: anterior, −5.6; lateral, 2.4; vertical, −9) with a specially designed microdialysis probe that allows the direct infusion of large peptides within the sampling area (Navarro et al., 2015). Some animals were implanted simultaneously with two regular probes, one in the VTA and the second one in the ipsilateral shell of the nucleus accumbens (NAc; coordinates in millimeters from bregma: anterior, 1.6; lateral, 0.5; vertical, −5.1) with a regular microdialysis probe. After surgery, rats were allowed to recover in hemispherical cages (model CMA 120, CMA Microdialysis) equipped with two-channel overhead fluid swivels (Instech) connected to a sample collector (model CMA 470, CMA Microdialysis). Twenty-four hours after implanting the probes, experiments were performed on freely moving rats in the same hemispherical home cages in which they recovered overnight from surgery. An artificial CSF (ACSF) containing (in mm) 144 NaCl, 4.8 KCl, 1.7 CaCl_2_, and 1.2 MgCl_2_ was pumped through the probe at a constant rate of 1 µl/min. After a washout period of 90 min, dialysate samples were collected at 20 min intervals. For peptide infusion, ghrelin and the liver expressed antimicrobial peptide 2 (LEAP2), an endogenous antagonist of GHS-R1a (Ge et al., 2018), and were dissolved in ACSF to a final concentration of 10 μm. The peptides were injected with a 1 µl syringe (Hamilton) driven by an infusion pump and coupled with silica tubing (inner diameter, 73 µm; Polymicro) to the microdialysis probe infusion cannula (dead volume, 40 nl), which was primed with ACSF and plugged during implantation and delivered at a rate of 16.6 nl/min. The GHS-R1a antagonist YL781, the D1R/D5R antagonist SCH23390, and the protein kinase A (PKA) inhibitor H-89 were administered within the VTA by constant perfusion by reverse dialysis within the VTA (all at 10 μm). At the end of the experiment, rats were given an overdose of Equithesin, the brains were extracted and fixed in formaldehyde, and probe placement was verified using cresyl violet staining. Dopamine content was measured by HPLC coupled with a coulometric detector (5200a Coulochem III, ESA).

### Patch-clamp electrophysiology.

Horizontal slices containing the VTA (225 μm) were prepared as described in detail previously (Kotecki et al., 2015) from 6- to 8-week-old Pitx3-eGFP(+) or C57BL6/J mice using previously identified putative dopaminergic neuron characteristics (Cruz et al., 2004; Arora et al., 2011). Results did not differ from those in EGFP^+^ cells and thus were combined. Current-clamp recordings were performed at 32–34°C using borosilicate electrodes (2.5–4.5 MΩ) filled with a K-gluconate solution (Hearing et al., 2013; Anderson et al., 2019) to assess spontaneous activity (*I* = 0). Upon achieving whole cell, cells were permitted to stabilize over a 2–5 min period before drug application. Drugs were bath applied using a gravity perfusion system at a rate of 2–2.5 ml/min. Each drug was applied for at least 5 min, with measures of frequency taken only after at least three consecutive sweeps of stable firing was obtained. For studies involving receptor antagonists (eticlopride, SCH39166, JMV2959), drugs were applied before and during subsequent application of ghrelin. Drug concentrations were as follows: ghrelin, 500 nm; SCH39166, 10 μm; JMV2959, 10 μm; and eticlopride, 2 μm.

### Statistics.

Parametric statistics (paired or nonpaired *t* test, one-way, bifactorial, or repeated-measures ANOVA followed by Dunnett's or Tukey's multiple-comparison test) were used, since the different groups analyzed showed normality and homogeneity of variance. For patch-clamp electrophysiological experiments, paired *t* test or repeated-measures ANOVA were used because measures (neural spike firing) were taken two or three times (baseline vs postdrug vs postdrug combinations) within the same cell to determine whether the mean difference between the repeated observations is zero. Details of the statistical analysis for each experiment, statistical test, sample size, and degree of significance are described in the corresponding figure legend. GraphPad Prism software version 7 was used for the statistical analysis.

## Results

### GHS-R1b-dependent oligomerization of GHS-R1a with D5R

The biophysical techniques Bioluminescence Resonance Energy Transfer BRET^1^ and BRET^2^ and SRET were performed in HEK293T cells transfected with different combinations of D5R fused to RLuc (D5R-RLuc), GHS-R1a fused to YFP (GHS-R1a-YFP), or GFP^2^ (GHS-R1a-GFP^2^) and either nonfused GHS-R1b or GHS-R1b fused to YFP (GHS-R1b-YFP). In BRET^1^ and BRET^2^, RLuc was used as the donor chromophore, but with two different RLuc substrates, coelenterazine H and DeepBlueC, which induce two different bioluminescent light emissions (485 and 410 nm, respectively), allowing energy transfer to a close YFP or GFP^2^ acceptor chromophore, respectively. These acceptors emit 530 nm (YFP) or 510 nm (GFP^2^) light, which can either be detected (for the respective BRET^1^ or BRET^2^ measurements) or result in a FRET to a second acceptor chromophore (for SRET measurements). In SRET, YFP was used as the second acceptor on transfer of energy from GFP^2^, which was the first acceptor from DeepBlueC-induced Rluc bioluminescence (for detailed description of techniques, see Carriba et al., 2008).

**Figure 1.**
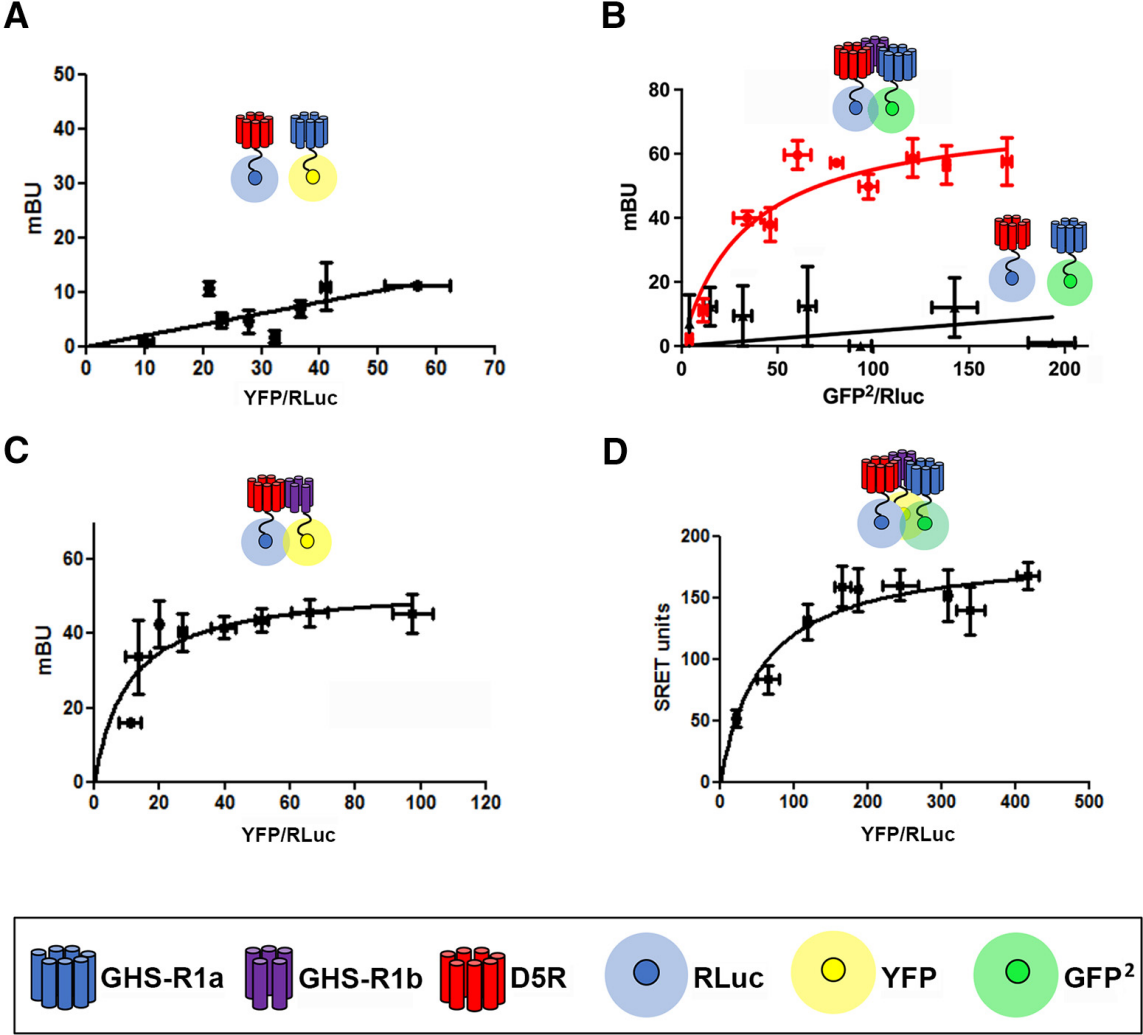
GHS-R1a:GHS-R1b:D15R oligomerization in HEK293T cells. ***A***, BRET^1^ saturation experiment, showing a straight line, in cells cotransfected with D5R-RLuc cDNA (0.8 µg) and increasing amounts of GHS-R1a-YFP (0.25–3 µg). ***B***, BRET^2^ saturation experiments in cells cotransfected with D5R-RLuc cDNA (0.8 µg) and increasing amounts of GHS-R1a-GFP^2^ cDNA (0.25–2 µg) in the presence (red saturation curve) and absence (black straight line) of cotransfected nonfused GHS-R1b (0.3 µg). ***C***, BRET^1^ saturation experiment showing a saturation curve in cells cotransfected with D5R-RLuc cDNA (0.8 µg) and increasing amounts of GHS-R1b-YFP (0.1–1.5 µg). ***D***, SRET saturation experiment showing a SRET saturation curve in cells cotransfected with a constant amount of GHS-D5R-RLuc cDNA (1 µg) and GHS-R1a-GFP^2^ cDNA (1.5 µg), and increasing amounts of GHS-R1b-YFPcDNA (0.1–1.5 µg). The relative amounts of BRET or SRET are given as a function of 100× the ratio between the fluorescence of the acceptor and the luciferase activity of the donor. BRET and SRET values are expressed as mBU or mSU and are given as the mean ± SD of six to eight experiments grouped as a function of the amount of BRET or SRET acceptor.

Saturation BRET^1^, BRET^2^, and SRET experiments were performed to support a specific interaction between the receptors fused to the corresponding chromophores from a nonspecific random collision. Energy transfer was then determined using the same transfected quantities of the receptor fused to the donor (and the first acceptor in SRET experiments) versus increasing quantities of the receptor fused to the acceptor chromophore (or second acceptor in SRET experiments; Carriba et al., 2008). Under the present experimental conditions, a BRET saturation curve, indicative of oligomerization, could be obtained with BRET^1^ experiments in cells transfected with D5R-RLuc and GHS-R1b-YFP (Fig. 1*C*), but not with GHS-R1a-YFP, which showed a straight line, indicative of nonspecific random collision (Fig. 1*A*). The same lack of saturation was also observed with BRET^2^ experiments in cells transfected with D5R-RLuc and GHS-R1a-GFP^2^ (Fig. 1*B*). Nevertheless, a clear saturation curve was obtained when the cells were cotransfected with nonfused GHS-R1b (Fig. 1*B*). In the same way, a clear saturation curve was obtained in SRET experiments in cells transfected with D5R-RLuc, GHS-R1a-GFP^2^, and GHS-R1b-YFP (Fig. 1*D*). These results indicate that, the same as with D1R (Navarro et al., 2016), GHS-R1b facilitates oligomerization of D5R with GHS-R1a. Since GHS-R1b, but not GHS-R1a, oligomerizes with D5R, these results strongly suggest that GHS-R1b establishes the link between D5R and GHS-R1a in the GHS-R1a:GHS-R1b:D5R oligomer.

### Differences between the signaling of GHS-R1a:GHS-R1b:D1R and GHS-R1a:GHS-R1b:D5R oligomers in mammalian transfected cells

**Figure 2.**
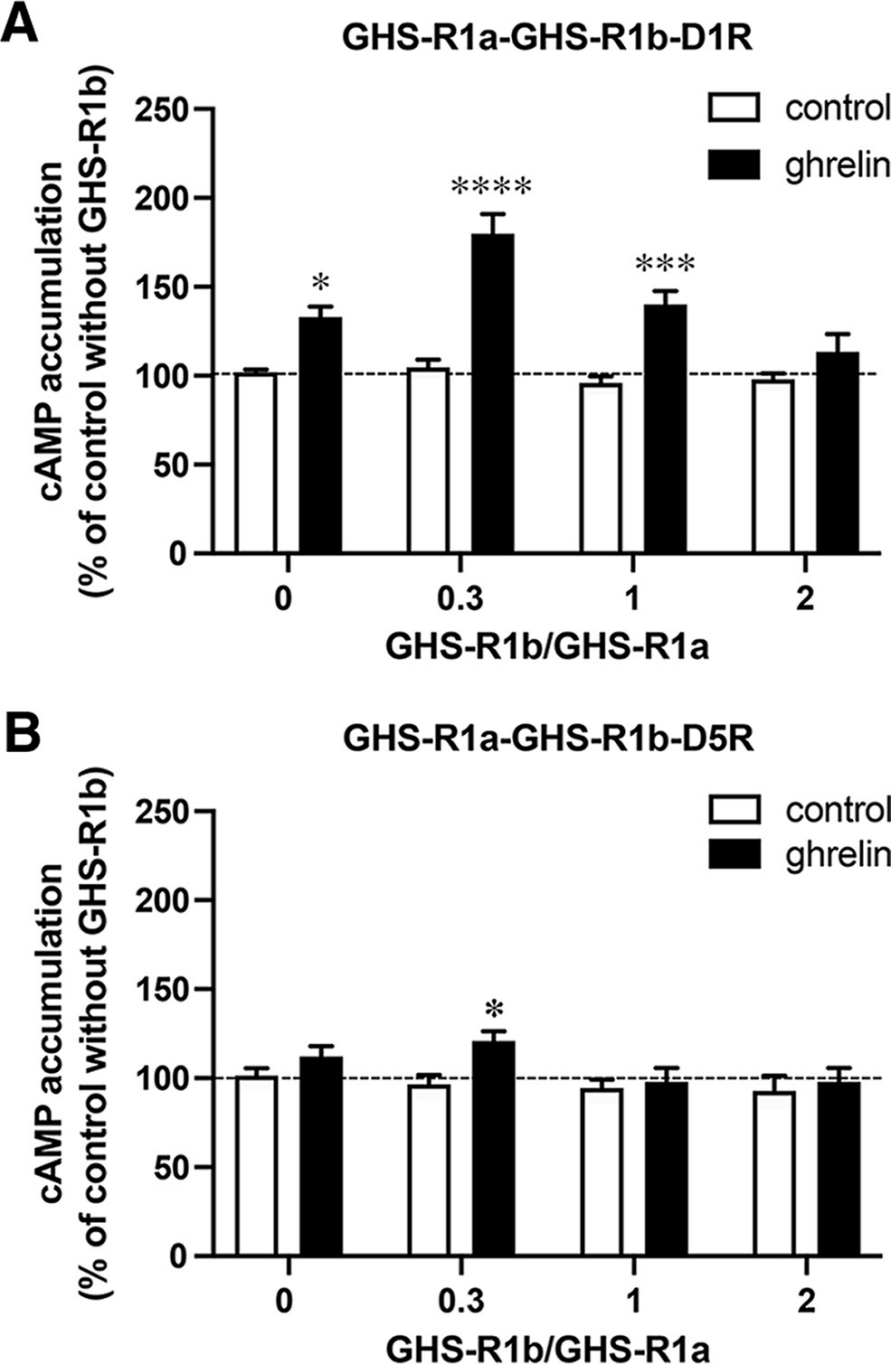
Analysis of cAMP accumulation in HEK293T cells expressing GHS-R1a, GHS-R1b, and D1R or GHS-R1a, GHS-R1b, and D1R. ***A***, Cells were transfected with GHS-R1a cDNA (1 µg) and D1R cDNA (1 µg) in the presence of increasing amounts of GHS-R1b cDNA (0, 0.3, 1, or 2 µg). ***B***, Cells were transfected with GHS-R1a cDNA (1 µg) and D5R cDNA (1 µg) in the presence of increasing amounts of GHS-R1b cDNA (0, 0.3, 1, or 2 µg); cAMP levels were analyzed after the addition of ghrelin (50 nm) or vehicle and are expressed as the percentage of cells treated with vehicle and not transfected with GHS-R1b (broken line), and are represented as the mean ± SEM of six experiments performed in triplicate. In ***A***, a bifactorial ANOVA demonstrates a significant effect of ghrelin (*p* < 0.0001) and GHS-R1b (*p* < 0.0001); significantly different versus the respective vehicle-treated control group: *p < 0.05, ***p < 0.001, and ***p < 0.0001, respectively; Tukey's multiple-comparison test. In ***B***, a bifactorial ANOVA demonstrates a significant effect of ghrelin (*p* < 0.05) without a GHS-R1b effect; significantly different versus the respective vehicle-treated control group: *p < 0.05; Tukey's multiple-comparison test.

The inability of GHS-R1b to bind ghrelin and signal indicates that any differential interaction between GHS-R1a and D1R/D5R ligands that depends on the presence of GHS-R1b is dependent on the formation of GHS-R1a:GHS-R1b:D1R or GHS-R1a:GHS-R1b:D5R oligomers. In our previous study, both D1R/D5R agonists and ghrelin were able to produce a Gs-dependent cAMP formation upon GHS-R1a:GHS-R1b:D1R oligomerization (Navarro et al., 2016). The analysis of cAMP formation showed that coadministration of ghrelin and the D1R/D5R agonist SKF81297 did not produce an additive effect, indicative of negative cross talk (Navarro et al., 2016). Also dependent on GHS-R1a:GHS-R1b:D1R oligomerization, the D1R/D5R antagonist SCH23390 counteracted the effects of both SKF81297 and ghrelin on cAMP formation. In addition, the GHS-R1a antagonist YIL781 also counteracted the effects of both ghrelin and SKF81297. In the present study, we also analyzed the effect of ghrelin on cAMP formation in HEK293T cells cotransfected with GHS-R1a and D1R cDNA and increasing amounts of GHS-R1b cDNA (Fig. 2*A*), with parallel experiments in cells cotransfected with GHS-R1a, D5R, and increasing amounts of GHS-R1b (Fig. 2*B*). The results from cells transfected with the D1R reproduced our previous finding of a ghrelin-induced cAMP accumulation facilitated by cotransfection with GHS-R1b (Navarro et al., 2016). Also, as previously shown, the effect of ghrelin (100 nm) depended on the GHS-R1b/GHS-R1a cDNA ratio, following an inverted u-shaped curve, with an increase of 26%, 72%, 46% and 15% versus control values at cDNA ratios of 0, 0.3, 1, and 2, respectively (Fig. 2*A*). Different from our previous studies (Navarro et al., 2016; Casanovas et al., 2021), the small effect of ghrelin observed in the absence of GHS-R1b reached statistical significance (Fig. 2*A*). This would agree with GHS-R1a being still able to induce a certain degree of activation of Gs when cotransfected with D1R and in the absence of GHS-R1b. On the other hand, ghrelin (100 nm) was practically ineffective at promoting cAMP accumulation when cotransfected with GHS-R1b and D5R and only showed a small but significant effect (26% increase vs control) at a GHS-R1b/GHS-R1a ratio of 0.3 (Fig. 2*B*).

**Figure 3.**
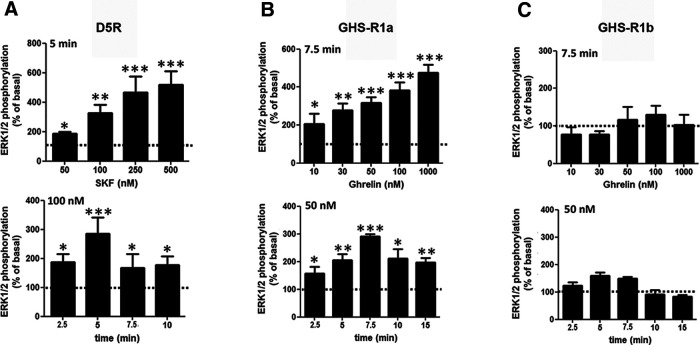
Analysis of ligand-induced ERK1/2 phosphorylation in HEK293T cells expressing D5R, GHS-R1a, or GHS-R1b. ***A***, Cells were transfected with D5R cDNA (1 µg) and treated with increasing concentrations of SKF81297 (50–500 nm) for 5 min (top graph) or for different times (2.5–10 min) with 100 nm SKF81297 (bottom graph). ***B***, ***C***, Cells were transfected with GHS-R1a or GHS-R1b cDNA (1 µg in both cases) and treated with increasing concentrations of ghrelin (10–1000 nm) for 7.5 min (top graphs) or for different times (2.5–15 min) with 50 nm ghrelin (bottom graphs). Phosphorylated ERK1/2 levels are expressed as a percentage of nontreated cells (broken line) and are represented as the mean ± SEM of five different experiments performed in duplicate; significantly different versus nontreated cells: *p < 0.05, **p < 0.01, and ***p < 0.001, respectively; one-way ANOVA followed by Tukey's multiple-comparison test.

**Figure 4.**
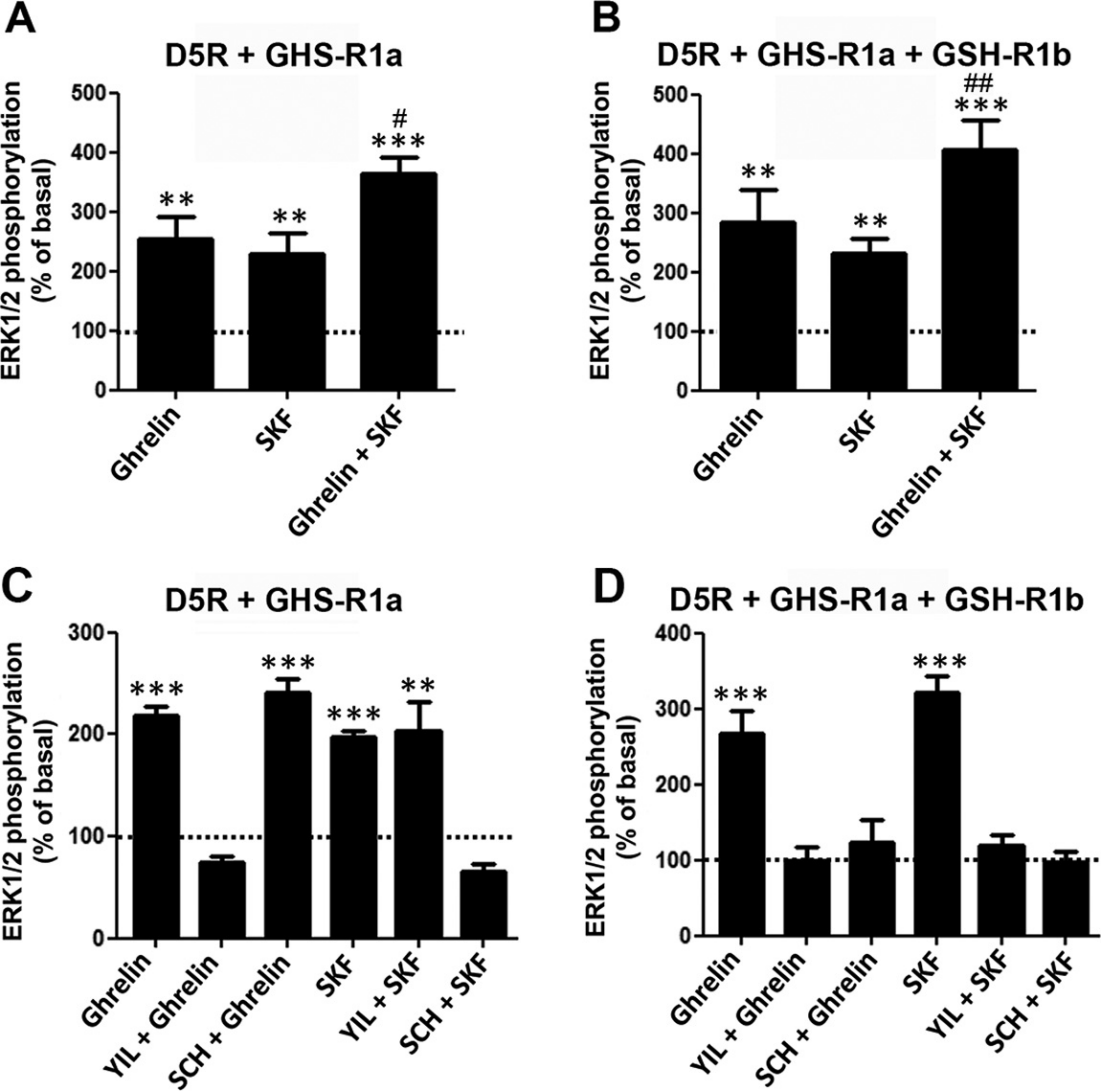
Comparison of ligand-induced ERK1/2 phosphorylation in HEK293T cells expressing D5R and GHS-R1a with or without GHS-R1b. ***A***, ***C***, Cells were transfected with D5R and GHS-R1a cDNA (1 µg in both cases). ***B***, ***D***, Cells were transfected with D5R cDNA (1 µg), GHS-R1a cDNA (1 µg), and GHS-R1b cDNA (0.3 µg). In ***A*** and ***B***, cells were treated for 7 min with ghrelin (50 nm), the D1R/D5R agonist SKF81297 (100 nm) or both compounds. In ***C*** and ***D***, cells were also treated for 7 min with either ghrelin (50 nm) or SKF81297 (100 nm), after previous treatment for 10 min with the GHS-R1a antagonist YL781 (1 μm) or the D1R/D5R antagonist SCH23390 (1 μm). Phosphorylated ERK1/2 levels are expressed as a percentage of nontreated cells (broken line) and are represented as the mean ± SEM of six to eight different experiments performed in duplicate; significantly different versus cells only treated with SKF81297: **#***p* < 0.05 and **##***p* < 0.01, respectively; one-way ANOVA followed by Tukey's multiple-comparison test; significantly different versus nontreated cells: **p < 0.01 and ***p < 0.001, respectively; one-way ANOVA followed by Tukey's multiple-comparison test.

**Figure 5.**
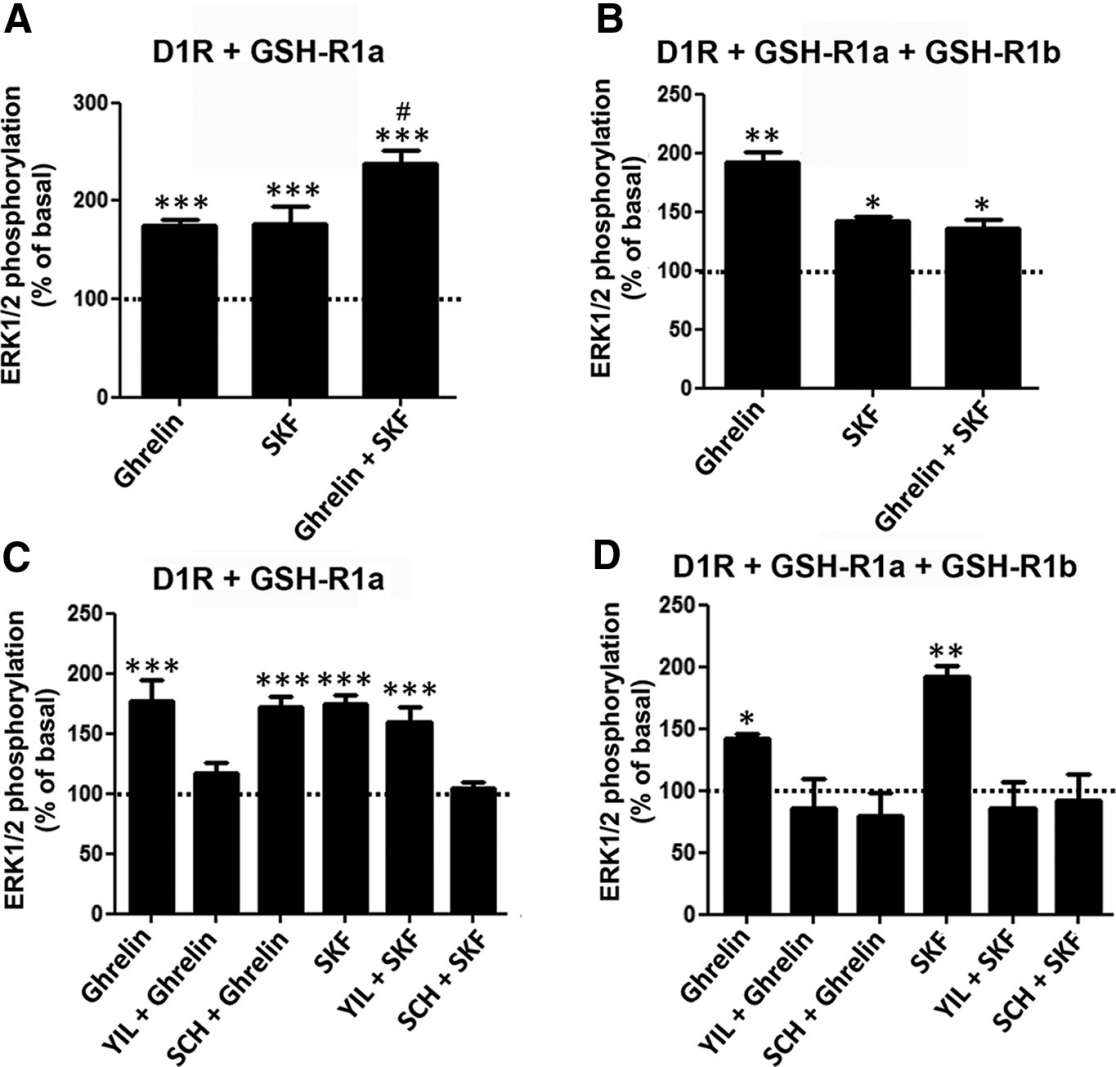
Comparison of ligand-induced ERK1/2 phosphorylation in HEK293T cells expressing D1R and GHS-R1a with or without GHS-R1b. ***A***, ***C***, Cells were transfected with D1R and GHS-R1a cDNA (1 µg in both cases). ***B***, ***D***, Cells were transfected with D1R cDNA (1 µg), GHS-R1a cDNA (1 µg), and GHS-R1b cDNA (0.3 µg). In ***A*** and ***B***, cells were treated for 7 min with ghrelin (50 nm), the D1R/D5R agonist SKF81297 (100 nm), or both compounds. In ***C*** and ***D***, cells were also treated for 7 min with either ghrelin (50 nm) or SKF81297 (100 nm) after previous treatment for 10 min with the GHS-R1a antagonist YL781 (1 μm) or the D1R/D5R antagonist SCH23390 (1 μm). Phosphorylated ERK1/2 levels are expressed as a percentage of nontreated cells (broken line) and are represented as the mean ± SEM of six to eight different experiments performed in duplicate); significantly different versus cells only treated with SKF81297: **#***p* < 0.05; one-way ANOVA followed by Tukey's multiple-comparison test; *, ** and ***: significantly different versus nontreated cells: *p < 0.05, **p < 0.01, and ***p < 0.001, respectively; one-way ANOVA followed by Tukey's multiple-comparison test.

If comparable, the results with cAMP accumulation would indicate a clear difference in the functional properties of the D_1_-like receptors on oligomerization with GHS-R1a and GHS-R1b, with D1R, but not D5R, increasing the ability of ghrelin to signal through Gs and activate AC. Therefore, the same cells (HEK293T) and transfection methodology (amount of D1R or D5R cDNAs as well as GHS-R1a and GHS-R1b cDNAs, at a GHS-R1b/GHS-R1a ratio of 0 or 0.3) was applied to compare differences between GHS-R1a:GHS-R1b:D1R and GHS-R1a:GHS-R1b:D5R oligomers on MAPK signaling, which does not necessarily depend on Gs activation. We first analyzed the effect of different concentrations and incubation times of ghrelin and SKF81297 in HEK293T cells transfected with either D5R (Fig. 3*A*) GHS-R1a (Fig. 3*B*), or GHS-R1b (Fig. 3*C*) on ERK1/2 phosphorylation. Fused receptors were used to demonstrate their functionality. As expected, ghrelin was effective in cells transfected with GHS-R1a (Fig. 3*B*), but not with GHS-R1b (Fig. 3*C*). From the results of the different agonist concentrations and time exposures (Fig. 3), 100 nm SKF81297 and 50 nm ghrelin and a time of incubation of 7 min were selected as most appropriate to analyze the effect of different ligand interactions on MAPK signaling in cells cotransfected with GHS-R1a with or without GHS-R1b and D5R (Fig. 4) or D1R (Fig. 5). In both cases, SKF81297 and ghrelin were effective and produced the same effect in the absence (Figs. 4*A*, 5*A*) or presence of GHS-R1b (Figs. 4*B*, 5*B*). Remarkably, ghrelin was very effective in cells transfected with D5R, indicating that the scarce activating effect of AC in cells cotransfected with GHS-R1a, GHS-R1b, and D5R versus those cotransfected with GHS-R1a, GHS-R1b, and D1R was not dependent on a general reduction of D5R function, but on the selective ability of the D1R to promote a ghrelin-mediated Gs activation in the GHS-R1a:GHS-R1b:D1R oligomer.

The comparison of the compound effect of ghrelin and SKF81297 uncovered another pharmacodynamic difference that depends on the D_1_-like receptor subtype. Only in cells cotransfected with GHS-R1a, GHS-R1b, and D1R was there evidence for negative cross talk on coadministration of ghrelin and SKF81297 (Fig. 5*B*). On the other hand, negative cross talk was not observed in cells cotransfected with GHS-R1a, GHS-R1b, and D5R, where coadministration of both agonists produced an additive response (with the effect of the compound administration being significantly higher than that of SKF81297 alone; Fig. 4*B*). These results would also complement the previously reported selective negative cross talk on AC signaling in cells cotransfected with GHS-R1a, GHS-R1b, and D1R, but not in cells not cotransfected with GHS-R1b, indicating its dependence on GHS-R1a:GHS-R1b:D1R oligomerization (Navarro et al., 2016).

As an orthogonal measure from the negative cross talk between agonists, a strong cross-antagonism of the D1R/D5R antagonist SCH23390 and the GHS-R1a antagonist YL781 (Esler et al., 2007) could be demonstrated in cells cotransfected with either D5R or D1R and GHS-R1a, but only when also cotransfected with GHS-R1b (compare Figs. 4*D*, 5*D*, and 4*C*, 5*C*). Thus, in cells expressing D5R and GHS-R1a, but in the absence of GHS-R1b, the ability of ghrelin to activate GHS-R1a and produce ERK1/2 phosphorylation could only be counteracted by the GHS-R1a antagonist YL781 (1 μm; Fig. 4*C*), while it could also be counteracted by the D1R/D5R antagonist SCH23390 (1 μm) in the presence of GHS-R1b (Fig. 4*D*). Likewise, the ability of the D1R/D5R agonist SKF81297 to activate MAPK signaling could only be counteracted by SCH23390 in the absence of GHS-R1b (Fig. 4*C*) and was also counteracted by YIL781 in the presence of GHS-R1b (Fig. 4*D*). This same pattern was observed when D1R was present in the oligomeric complex, instead of D5R (Fig. 5*C*,*D*). Thus, the presence of GHS-R1b induces the ability of GHS-R1a and D1R/D5R antagonists to respectively cross-antagonize D1R/D5R and GHS-R1a agonists, which is consistent with its proposed role in supporting oligomerization between GHS-R1a and D1R or D5R.

### MAPK signaling of GHS-R1a:GHS-R1b:D1R oligomers in the VTA

**Figure 6.**
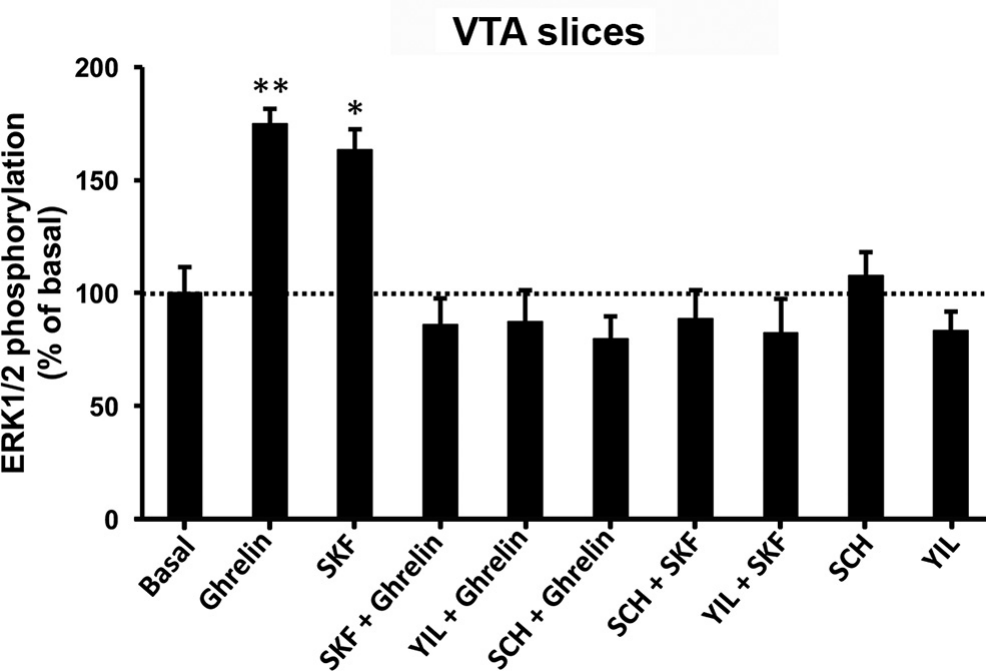
Analysis of ligand-induced ERK1/2 phosphorylation in rat VTA slices. ERK1/2 phosphorylation was determined in rat VTA slices pretreated for 3 h with medium. Slices were then incubated for 20 min with medium, the GHS-R1a antagonist YIL781 (5 μm), or the D1R/D5R antagonist SCH23390 (5 μm), and were treated for 12 min with medium, ghrelin (500 nm), the D1R/D5R agonist SKF81297 (500 nm), or both. Phosphorylated ERK1/2 levels are expressed as a percentage of nontreated slices (broken line) and are represented as the mean ± SEM of four to five slices; significantly different versus nontreated slices: *p < 0.05 and **p < 0.01, respectively; one-way ANOVA followed by Tukey's multiple-comparison test.

Ghrelin and SKF81297-induced ERK1/2 phosphorylation were then evaluated in VTA slices of Sprague Dawley rats using a previously reported method that allowed the study of MAPK signaling of other GPCR complexes (Navarro et al., 2015; Moreno et al., 2017; see also Materials and Methods). As in previous studies, higher ligand concentrations were used compared with transfected cell preparations to allow sufficient penetration into the tissue. At 500 nm, both ghrelin and the D1R agonist SKF81297 produced a significant increase in ERK1/2 phosphorylation, but not when coadministered, which is indicative of a negative cross talk (Fig. 6). The effects of both agonists were counteracted by either antagonist, SCH23390 or YL781 (at 5 μm), which is indicative of cross-antagonism (Fig. 6). That this cross-antagonism represents a biochemical characteristic of GHS-R1a:GHS-R1b:D1R and GHS-R1a:GHS-R1b:D5R oligomers implies that either of them could represent a major population of functional GHS-R1a receptors in the VTA. In addition, to our knowledge, there are not available ligands that can discriminate between D1R and D5R, including SKF81297 and SCH23390. While not conclusive, the evidence of negative cross talk between ghrelin and SKF81297, which was specific for D1R in HEK293T cells, is more consistent with D1R as the predominant D_1_-like receptor forming functional complexes with GHS-R1a and GHS-R1b in the VTA.

### GHS-R1a:GHS-R1b:D1R oligomer-mediated modulation of dopaminergic neuronal function in the VTA

The local application of ghrelin in the rodent VTA has been previously shown to promote an increase in the activity of the dopaminergic neurons with an increase in their firing rate (Abizaid et al., 2006) and in dopamine release in the ipsilateral NAc (Jerlhag et al., 2007) using patch-clamp electrophysiology in brain slices and *in vivo* microdialysis, respectively. The lack of availability of specific antagonists hampered the demonstration of the specific involvement of GHS-R1a in these effects of ghrelin. Nevertheless, brain slices from GHSR KO mice were used in the electrophysiological experiments and were shown to be insensitive to the effect of ghrelin (Abizaid et al., 2006). Selective and potent small-molecule GHS-R1a competitive antagonists have now been developed, such as YIL781 (Esler et al., 2007) and JMV2959 (Moulin et al., 2007, 2013). In addition, an endogenous antagonist of GHS-R1a, LEAP2, has recently been discovered (Ge et al., 2018). LEAP2 is mainly produced in the liver and small intestine. Its secretion is suppressed by fasting and has been shown to block the effects of ghrelin *in vivo*, including food intake, growth hormone release, and maintenance of viable glucose levels during chronic caloric restriction (Ge et al., 2018). Although it was initially characterized as a negative allosteric modulator (Ge et al., 2018), subsequent studies indicate that it is an inverse agonist (M'Kadmi et al., 2019).

**Figure 7.**
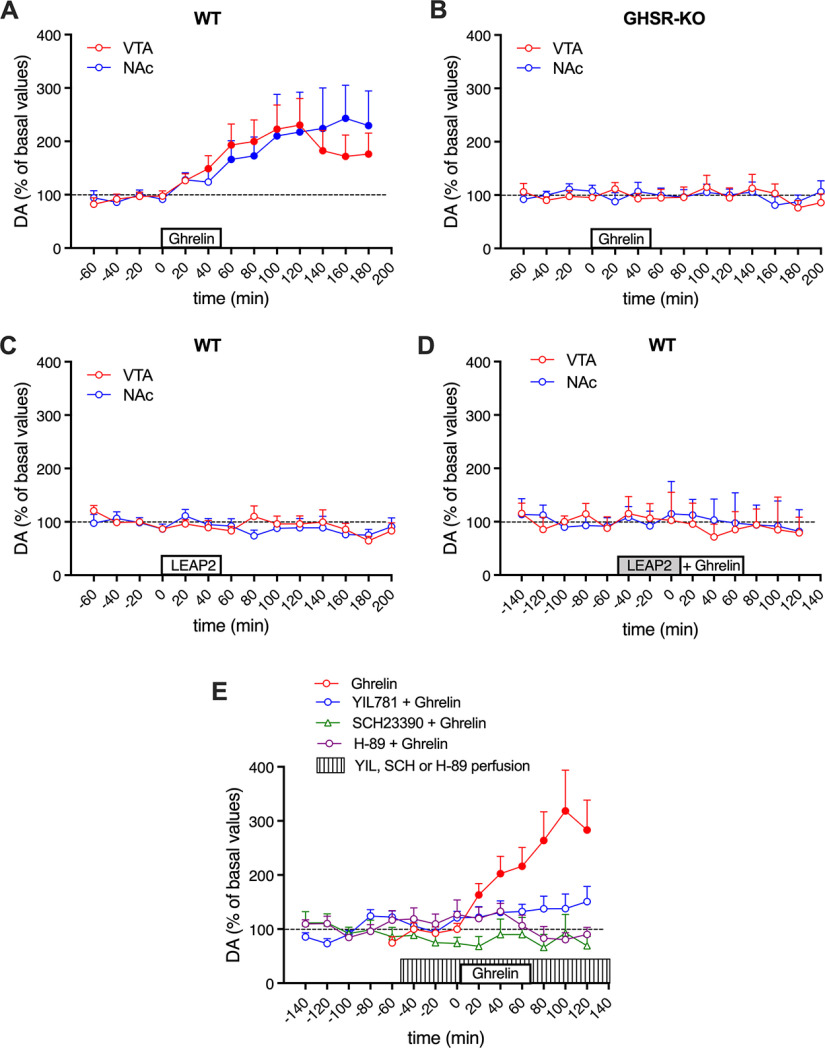
Activation of VTA dopaminergic cells by ghrelin: microdialysis experiments. ***A–D***, Somatodendritic and NAc dopamine release in GHS-R1 CRISPR/Cas9 KO Wistar rats (GHSR-KO) and WT; values represent dopamine concentrations from the VTA and the ipsilateral NAc, represented as the mean ± SEM (*n* = 7–14/group) of the percentage of baseline (average of three samples before infusion of ghrelin or LEAP2); white or gray rectangles on the *x*-axis: periods of infusion of ghrelin or LEAP2 (10 μm for both peptides). ***E***, Somatodendritic dopamine release in Sprague Dawley rats; values represent dopamine concentrations from the VTA, represented as the mean ± SEM (*n* = 7–14/group) of the percentage of baseline (average of three samples before infusion of ghrelin); white or lined rectangles on the *x*-axis: periods of infusion of ghrelin or perfusion (reverse dialysis) of the GHS-R1a antagonist YIL781, the D1R/D5R antagonist SCH23390, or the PKA inhibitor H-89 (10 μm for all compounds). Filled symbols represent significantly different means compared with the mean of the first three basal values (*p* < 0.05, repeated-measures ANOVA followed by Dunnett's multiple-comparison test).

By using a previously described *in vivo* microdialysis technique that allows the slow infusion of large peptides (Navarro et al., 2015; Moreno et al., 2017; Cai et al., 2019), we first investigated the ability of the local application of ghrelin and LEAP2, alone and in combination, to modulate the respective somatodendritic and terminal release of dopamine in the VTA and ipsilateral NAc. The recently developed and characterized GHS-R1a CRISPR/Cas9 KO Wistar rat and WT littermates (Zallar et al., 2019) were used to further demonstrate the dependence on GHS-R1a of the pharmacological effects of ghrelin and LEAP2. As previously reported by Jerlhag et al. (2007) with a bolus infusion (1 µg in 1 µl in 1 min), the slow local infusion of ghrelin (10 μm at 16 nl/ml) in the VTA of WT rats produced a significant increase in the extracellular levels of dopamine in the ipsilateral NAc (Fig. 7*A*). Furthermore, we demonstrate that ghrelin induces a significant somatodendritic dopamine release (Fig. 7*A*). This is expected since local dopamine release in the VTA is a correlate of dopaminergic cell firing, which is induced by the antidromic propagation of action potentials (Legault and Wise, 1999). On the other hand, somatodendritic dopamine release promotes activation of inhibitory autoreceptors of the dopamine D_2_ receptor subtype (D2-autoreceptors), with the consequent inhibition of cell excitability, limiting excessive neuronal activation (Adell and Artigas, 2004). As expected, ghrelin was ineffective in GHS-R KO rats, and no significant changes were observed with the extracellular levels of dopamine in the VTA or in the ipsilateral NAc (Fig. 7*B*). Less expected was the lack of effect of the VTA infusion of LEAP2 (10 μm) in the VTA or ipsilateral NAc of WT rats (Fig. 7*C*), since there is *in vitro* and *in vivo* evidence for a significant constitutive activity of GHS-R1a (Petersen et al., 2009; Damian et al., 2012; M'Kadmi et al., 2019). Nevertheless, coinfusion of LEAP2 completely counteracted the effect of ghrelin (both at 10 μm; Fig. 7*D*).

The effect of the slow local infusion of ghrelin on the somatodendritic dopamine release in the VTA was then evaluated in Sprague Dawley rats to compare with the efficacy of other previously reported neuropeptides using the same rat strain and methodology. At the same concentration (10 μm), ghrelin produced a much larger dopamine release (>200%; Fig. 6*E*) compared with orexin-A or endomorphin-1 (<100% in both cases; Navarro et al., 2015; Moreno et al., 2017). Constant perfusion by reverse dialysis within the VTA of the GHS-R1a antagonist YL781 (10 μm) or the D1R/D5R antagonist SCH23390 (10 μm) counteracted the dopamine-releasing effect of ghrelin (Fig. 7*E*). Finally, the perfusion of the PKA inhibitor H-89 (10 μm) also completely counteracted the effect of ghrelin (Fig. 7*E*). The cross-antagonism by SCH23390 supports that GHS-R1a:GHS-R1b:D_1_-like receptor oligomers localized in the VTA play a significant role in the modulation of the dopaminergic cell function. The inhibitory effect of the PKA inhibitor indicates a significant role of Gs–AC–cAMP signaling in the dopamine-releasing effect of ghrelin, which is again consistent with the interpretation that GHS-R1a:GHS-R1b:D1R, rather than GHS-R1a:GHS-R1b:D5R, oligomers represent the functionally dominant population of GHS-R1a receptors in the VTA.

However, the counteracting effect of the SCH23390 on ghrelin-induced somatodendritic dopamine release could just be an apparent cross-antagonism, since it has been previously shown that D_1_-like receptors localized in the VTA dopaminergic cells facilitate the desensitization of D2-autoreceptors on prolonged exposure to dopamine or D2R agonists (Nimitvilai and Brodie, 2010; Nimitvilai et al., 2012a, b), and the application of D1R/D5R antagonists can prevent this desensitization (Nimitvilai and Brodie, 2010). Another possible mechanism responsible for the counteracting effect of the SCH23390 could be its also demonstrated agonism on serotonin 5-HT_2C_ receptors (5-HT2CRs; Millan et al., 2001), which are localized in GABAergic and dopaminergic cells in the VTA (Bubar and Cunningham, 2007), where they appear to mediate an inhibitory influence of serotonin on dopamine neurotransmission (Bubar and Cunningham, 2006). Therefore, we also performed electrophysiologic measurements in identified VTA dopaminergic neurons, controlling the inhibitory role of D2-autoreceptors (with the addition of the D2R antagonist eticlopride) and using the selective D1R antagonist SCH39166, which does not bind to 5-HT2CR (Wamsley et al., 1991).

**Figure 8.**
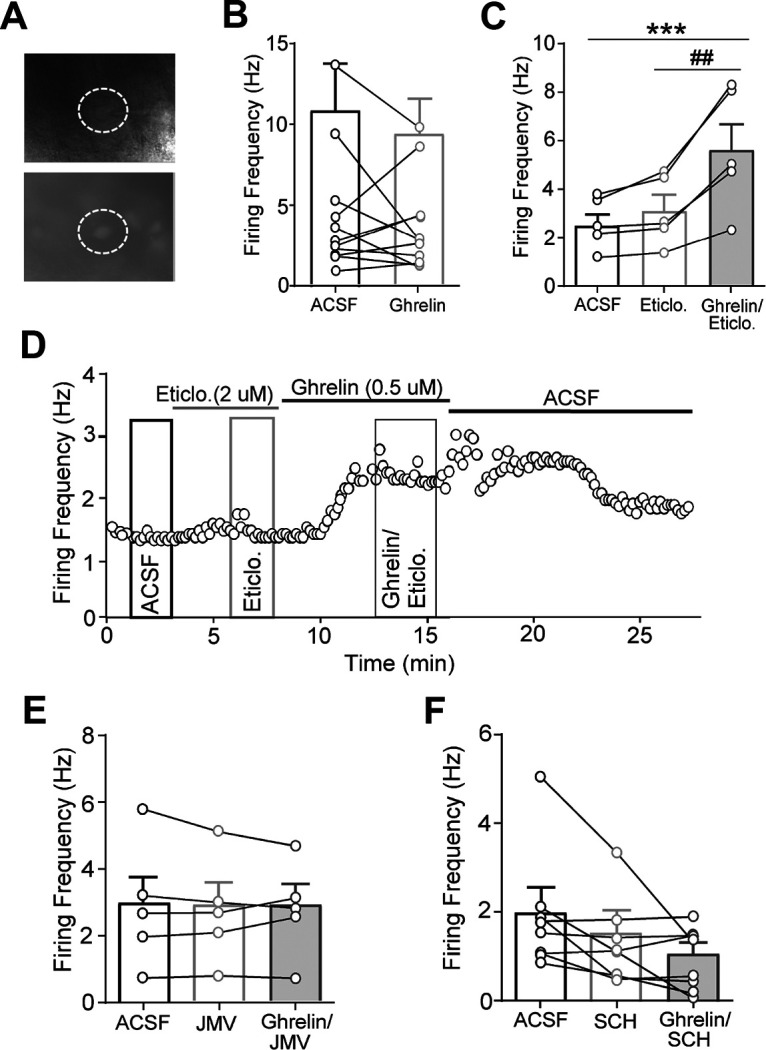
Activation of VTA dopaminergic cells by ghrelin: electrophysiological experiments. ***A***, eGFP-positive cell in the VTA of an acutely isolated brain slice from a Pitx3 mouse, as visualized via infrared microscopy (top) and epifluorescence (bottom); the dashed circle highlights the cell targeted for evaluation (pipette visible in the top). ***B***, Mean firing frequency of dopaminergic neurons in ACSF (baseline) and following 5 min bath application of ghrelin using a within-cell approach. ***C***, Mean firing frequency in ACSF and following bath application of the D2R antagonist eticlopride (5 min) and subsequent application of ghrelin and eticlopride. ***D***, Representative trace showing the impact of eticlopride and ghrelin in the presence of eticlopride on firing frequency followed by washout in ACSF; lines denote the duration of drug on/off periods, and highlighted boxes denote that the time data were collected and averaged for firing frequency in ACSF, eticlopride, and ghrelin plus eticlopride. ***E***, Mean firing frequency of dopaminergic neurons in the presence of ACSF, the GHS-R1a antagonist JMV2959, and ghrelin plus JMV2959. ***F***, Mean firing frequency in ACSF and following bath application of the D1R/D5R antagonist SCH39166 and subsequent application of ghrelin in the presence of SCH39166. ***p < 0.001 versus ACSF; ##*p* < 0.01 versus eticlopride alone, repeated-measures ANOVA followed by Tukey's multiple-comparison test.

We initially examined whether ghrelin influences the firing properties of VTA dopaminergic neurons with whole-cell patch-clamp recordings in C57BL6/J (WT) mice using classical electrophysiological properties to identify putative dopaminergic neurons. Because of the unexpected variability of the effect of ghrelin (increased or decreased firing), subsequent studies were performed in mice with C57BL6/J background expressing eGFP under the control of the promoter for the dopaminergic neuron-specific transcription factor Pitx3 (Pitx3-eGFP; Zhao et al., 2004; Fig. 8*A*). This genetic approach represents a more reliable method than classical electrophysiological measures for the identification of dopaminergic neurons, and there are numerous publications that have validated the use of Pitx3 mice to identify dopaminergic neurons (Labouèbe et al., 2007; Kotecki et al., 2015; McCall et al., 2017). In fact, VTA dopaminergic neurons are now known to be composed of a phenotypical heterogenous population of neurons that differ in their previously thought unique electrophysiological profile, which included the presence of a large hyperpolarization-activated cation current (*I*_h_), long action potential durations, narrow range of basal firing frequency, and a capacity for inhibition by D2R agonists, related to D2 autoreceptor-mediated feedback inhibition via somatodendritic dopamine release (Ford et al., 2006; Lammel et al., 2008; Margolis et al., 2008; Morales and Margolis, 2017). Only one of those measures, *I*_h_, was used to identify dopaminergic neurons in the study by Abizaid et al. (2006), which showed a significative ghrelin-induced increase in action potential frequency in putative dopaminergic cells from rat and mouse VTA.

Spontaneous action potentials were recorded in current clamp (*I* = 0). For the initial assessment of the effect of ghrelin, the baseline firing frequency in cells collected from C57BL6/J mice (WT; *n* = 6) and Pitx3 mice (*n* = 5; Fig. 8*B*) did not differ significantly (WT, 3.47 ± 1.3 Hz; Pitx3, 5.40 ± 2.1 Hz; nonpaired *t* test, *p* = 0.412). These data were therefore combined to illustrate the variable effect of ghrelin. Following at least 3–5 min of stable recording of action potentials, 0.5 μm ghrelin was applied via bath perfusion. Within-cell comparisons initially showed that while 10 of 11 cells exhibited a significant change in firing frequency (>20% change), there was no significant effect of ghrelin on action potential frequency (Fig. 8*B*; paired *t* test, *p* = 0.49). Subsequent examination of within-cell effects of ghrelin compared with baseline revealed that five cells exhibited an increase in firing frequency, while five showed a reduction in firing rate. *Post hoc* analysis of these two populations showed a mean 65% increase in firing frequency compared with baseline (baseline, 2.45 ± 0.55 Hz; ghrelin, 4.28 ± 1.2 Hz; paired *t* test, *p* = 0.052), whereas five showed a mean 47% reduction in firing (baseline, 6.75 ± 2.13; ghrelin, 3.64 ± 1.60; paired *t* test, *p* = 0.034). This differential effect of ghrelin could depend on the already established existence of two subsets of VTA dopaminergic neurons, with different sensitivity to D2-autoreceptor-mediated inhibition. The subset less sensitive to D2-autoreceptor inhibition is species dependent and seems to be mostly represented by dopaminergic cells projecting to the amygdala and to the frontal cortex in rats and mice, respectively (for review, see Morales and Margolis, 2017). As reductions in firing were often preceded by acute elevations in activity (data not shown), it was possible that ghrelin-induced increases in activity and associated somatodendritic release could drive a subsequent inhibition of activity in the subset of dopaminergic neurons more sensitive to D2-autoreceptor-mediated inhibition. To determine whether ghrelin-induced reductions in firing observed in a subset of cells reflects a D2R-dependent pause in firing (Beckstead et al., 2004; Beckstead and Williams, 2007; Ford et al., 2010; Bello et al., 2011; Courtney et al., 2012), we applied the D2R antagonist eticlopride before and in combination with ghrelin (Fig. 8*C*,*D*). Bath application of eticlopride alone slightly elevated firing rates compared with ACSF baseline (Fig. 8*C*). However, coapplication of eticlopride and ghrelin led to a significant increase in firing in five of five cells (repeated-measures ANOVA, *p* < 0.001; Fig. 8*C*) with a mean increase of 81 ± 0.8% compared with eticlopride alone (*p* < 0.001) and 115 ± 0.8% compared with ACSF (*p* = 0.002; Fig. 8*C*,*D*). Firing frequency did not differ between ACSF and eticlopride (*p* = 0.495).

Finally, we investigated whether GHS-R1a formation of oligomers with GHS-R1b and D_1_-like receptors mediated the observed ghrelin-induced increases in dopaminergic neuron firing. Baseline recordings were taken, then the GHS-R1a antagonist JMV2959 or the D1R/D5R antagonist SCH39166, plus eticlopride, were applied either alone or followed by the application of ghrelin. Repeated-measures ANOVA revealed no differences in firing frequency following the application of JMV2959 alone or JMV2959 plus ghrelin compared with ACSF (plus eticlopride; *p* = 0.83; Fig. 8*E*). Thus, similar to LEAP2 in the microdialysis experiments, JMV2959 did not behave as an inverse agonist on dopaminergic cell activity, although it has been described as an inverse agonist in some *in vitro* assays (Ramirez et al., 2019). This observation seems to disclose another property of GHS-R1a:GHS-R1b:D1R oligomers: a decrease in the constitutive activity of GHS-R1a. The same as for JMV2959, the effect of SCH39166 alone or plus ghrelin did not differ from ACSF baseline (plus eticlopride; *p* = 0.07; Fig. 8*F*). Together, these findings indicate that ghrelin augments firing frequency in dopaminergic neurons that are both sensitive and insensitive to D2R inhibition and that these actions are probably predominantly mediated by GHS-R1a:GHS-R1b:D1R oligomers.

## Discussion

The present study supports that GHS-R1a:GHS-R1b:D1R oligomers are the main mediators of the excitatory effects of ghrelin on VTA dopaminergic cells. We previously showed in mammalian transfected cells and in striatal neurons in culture that GHS-R1b facilitates oligomerization of GHS-R1a with D1R, switching GHS-R1a from its canonical Gq-mediated signaling to a Gs–AC–cAMP signaling (Navarro et al., 2016). As a biochemical signature of the GHS-R1a:GHS-R1b:D1R oligomer, a D1R/D5R antagonist could block ghrelin-induced cAMP formation in both cell preparations (Navarro et al., 2016). In the present study, the ghrelin-induced increase in the activity of VTA dopaminergic cells was found both to be dependent on Gs–AC–cAMP–PKA signaling and counteracted by D1R/D5R antagonists.

Our initial hypothesis was that D5R should be the principal D_1_-like receptor involved, in view of its established predominant expression in the mesencephalic dopaminergic cells in the rodent and human and nonhuman primates (Ciliax et al., 2000; Khan et al., 2000). In fact, the immunohistochemical results by Jiang et al. (2006), showing a significant colocalization of GHS-R1a and D1R in the mouse substantia nigra and VTA contradicted the consistently reported lack of D1R mRNA expression in the mesencephalic cells of the mammalian brain (Mansour et al., 1991; Mengod et al., 1991; Hurd et al., 2001). We first demonstrated that, similarly to D1R, D5R can form oligomers with GHS-R1a and GHS-R1b. With resonance energy transfer techniques in mammalian transfected cells, it was possible to show that D5R can form a complex with GHS-R1a, but only in the presence of GHS-R1b, which alone could also oligomerize with D5R. This strongly suggests that GHS-R1b acts as a link between D5R and GHS-R1a in the GHS-R1a:GHS-R1b:D5R complex, in view of the well established ability of GHS-R1b to oligomerize with GHS-R1a (see Introduction).

Significantly, we could find specific differences in the pharmacological properties of GHS-R1a:GHS-R1b:D1R and GHS-R1a:GHS-R1b:D5R oligomers. The pharmacological properties were dependent on the existence of the respective oligomeric complex and could be demonstrated by their dependence on the presence of GHS-R1b. Compared with the GHS-R1a:GHS-R1b:D1R oligomer, ghrelin promoted a weaker Gs–AC–cAMP signaling by the GHS-R1a:GHS-R1b:D5R oligomer. Nevertheless, MAPK signaling was pronounced with both oligomers and allowed us to disclose a second pharmacological difference. This is a negative cross talk between ghrelin and a D1R/D5R agonist within the GHS-R1a:GHS-R1b:D1R oligomer, but not the GHS-R1a:GHS-R1b:D5R oligomer. On the other hand, both oligomers showed cross-antagonism with MAPK signaling, with the D1R/D5R antagonist SCH23390 counteracting ghrelin and the GHS-R1a antagonist YL781 completely counteracting the effect of the D1R/D5R agonist SKF81297. Cross-antagonism has been repeatedly shown to be a specific property of GPCR heteromers (Ferré et al., 2014). That the presence of GHS-R1b induces the ability of GHS-R1a and D1R/D5R antagonists to respectively cross-antagonize D1R/D5R and GHS-R1a agonists, strongly argues for cross-antagonism between ghrelin and D1R/D5R ligands as a biochemical property of GHS-R1a:GHS-R1b:D1R or GHS-R1a:GHS-R1b:D5R oligomers. Although there are no available ligands that can differentiate pharmacologically from D1R and D5R, the present results disclose significant pharmacological differences between both receptors that depend on their oligomerization with GHS-R1a and GHS-R1b. These differences could then be used to identify the predominant D_1_-like receptor subtype forming oligomeric complexes with GHS-R1a and GHS-R1b, which modulate dopamine function within the VTA. However, it is important to point out that the evidence for the existence of GHS-R1b in the VTA is still correlational and that it needs still to be demonstrated.

Three different models were used to examine the activating effects of ghrelin in the VTA, as follows: (1) MAPK activation in VTA slices; (2) somatodendritic dopamine release in microdialysis experiments; and (3) increased activity of dopaminergic cells in patch-clamp experiments. The most pronounced effect was the increase of somatodendritic dopamine release in the VTA and its corresponding projecting area, the NAc, after a slow and sustained infusion of ghrelin (60 min) into the VTA. On the other hand, the electrophysiological experiments required D2-autoreceptor blockade to disclose the stimulatory effects of ghrelin. The most likely explanation for this difference is the reported desensitization of D2-autoreceptors after prolonged exposure to dopamine, which depends on costimulation of D_1_-like receptors, probably D5R localized in the VTA dopaminergic cells (Nimitvilai and Brodie, 2010; Nimitvilai et al., 2012a, b). The dependence on D1R/D5R activation, as well as on the activation of a Gq/PLC/PKC pathway and calcium signaling, suggests that a significant population of D2-autoreceptors could represent D2R-D5R heteromers (Rashid et al., 2007; So et al., 2009; Nimitvilai et al., 2012a, b). Inhibition of ghrelin-induced somatodendritic dopamine release by a D1R/D5R antagonist should therefore involve a reversal of autoreceptor sensitization. Nevertheless, the electrophysiological experiments demonstrated a clear cross-antagonism with a complete D1R/D5R antagonist-mediated blockade of ghrelin-induced activation of VTA dopaminergic cells on D2-autoreceptor blockade. These results implied that oligomers of GHS-R1a, GHS-R1b, and D_1_-like receptors could represent a main population of GHS-R1a within the VTA that modulates dopaminergic cell function.

Unexpectedly, negative cross talk was observed on MAPK activation in VTA slices induced by ghrelin and the D1R/D5R agonist SKF81297. In addition, the local perfusion in the VTA of a PKA inhibitor counteracted ghrelin-induced somatodendritic dopamine release, disclosing the pharmacological profile of the GHS-R1a:GHS-R1b:D1R oligomer. The lack of expression of D1R in the VTA dopamine neurons would therefore favor a presynaptic localization of the GHS-R1a:GHS-R1b:D1R oligomer in VTA glutamatergic or cholinergic excitatory inputs. Indeed, past work has shown that ghrelin-mediated increases in the firing frequency of putative VTA dopaminergic neurons are inhibited by blockade of AMPA- and NMDA-type glutamate receptors and align with elevations in the frequency of miniature excitatory postsynaptic currents—a phenomenon often equated with increased presynaptic glutamate release (Abizaid et al., 2006). Our results using patch-clamp electrophysiology are also compatible with a presynaptic effect of ghrelin, which may also explain its nonselective effect on those subsets of dopaminergic neurons that either do or do not express somatodendritic D2-autoreceptors.

There is evidence for a functional role of presynaptic D_1_-like receptors localized in glutamatergic terminals in the VTA, which when activated upon somatodendritic dopamine release promote glutamate release, and which seem to play an important role in the pharmacological effects of alcohol and psychostimulants (Kalivas and Duffy, 1995; Deng et al., 2009; Xiao et al., 2009). Cholinergic or glutamatergic neurons from the laterodorsal tegmental area (LDTg) constitute a major excitatory input to the VTA (Geisler et al., 2007; Watabe-Uchida et al., 2012), and the LDTg has been shown to express GHS-R1a mRNA (Guan et al., 1997; Zigman et al., 2006). To our knowledge, there is no evidence for D_1_-like receptor expression in the LDTg, but there are other brain areas that also provide significant glutamatergic inputs to the VTA and express both GHS-R1a and D1R, which could therefore be expressed in their VTA nerve terminals. Those include the paraventricular nucleus of the hypothalamus, the retrorubral field, and the lateral parabrachial nucleus (Thibaut et al., 1990; Guan et al., 1997; Rivkees and Lachowicz, 1997; Czyrak et al., 2000; Maltais et al., 2000; Zigman et al., 2006; Geisler et al., 2007; Coizet et al., 2010; Watabe-Uchida et al., 2012; Mani et al., 2014). Experiments are in progress to determine the possible existence of GHS-R1a:GHS-R1b:D1R oligomers in these VTA excitatory inputs, which should provide a better understanding of the mechanisms by which GHS-R1a signaling is able to influence the function of VTA dopaminergic neurons.

Apart from the localization of GHS-R1a:GHS-R1b:D1R oligomers within the VTA, the origin of the endogenous ghrelin involved in the functional activation of these receptor complexes needs still to be determined. Some of the proposed mechanisms by which endogenous ghrelin could activate GHS-R1 in the VTA include its local synthesis, its peripheral transit to the VTA, and an indirect activation through circuit-level effects (Cabral et al., 2017; Edwards and Abizaid, 2017; Perello et al., 2019; Wenthur et al., 2019). The evidence for central synthesis of ghrelin is modest and hotly debated (Cabral et al., 2017; Edwards and Abizaid, 2017). Nevertheless, we have recently demonstrated that cocaine-mediated reward is sensitive to systemically administered GHS-R1a antagonists, but not to sequestration of peripheral ghrelin with peripherally acting specific antibodies (Wenthur et al., 2019), which would argue against the transport of peripheral ghrelin to the CNS as the primary source of its facilitatory effects on reward-associated behaviors.

Irrespective of the specific origin of endogenous ghrelin, the present study strongly suggests that the GHS-R1a:GHS-R1b:D1R oligomer localized in the VTA can provide an important pharmacological target for alcohol and psychostimulant use disorders, in view of the preclinical and clinical evidence suggesting that GHS-R1a antagonists can be useful pharmacological approaches to treat in these disorders (Jerlhag et al., 2009, 2010; Zallar et al., 2017; Farokhnia et al., 2019; Wenthur et al., 2019; Edvardsson et al., 2021).
